# CD103^+^ CD8 T Cells in the *Toxoplasma*-Infected Brain Exhibit a Tissue-Resident Memory Transcriptional Profile

**DOI:** 10.3389/fimmu.2017.00335

**Published:** 2017-03-29

**Authors:** Tyler A. Landrith, Suhas Sureshchandra, Andrea Rivera, Jessica C. Jang, Maham Rais, Meera G. Nair, Ilhem Messaoudi, Emma H. Wilson

**Affiliations:** ^1^School of Medicine, University of California, Riverside, CA, USA

**Keywords:** CD103, neuroimmunology, chronic infection, *Toxoplasma gondii*, CD8^+^ T cell memory, tissue-resident memory cells

## Abstract

During chronic infection, memory T cells acquire a unique phenotype and become dependent on different survival signals than those needed for memory T cells generated during an acute infection. The distinction between the role of effector and memory T cells in an environment of persistent antigen remains unclear. Here, in the context of chronic *Toxoplasma gondii* infection, we demonstrate that a population of CD8 T cells exhibiting a tissue-resident memory (T_RM_) phenotype accumulates within the brain. We show that this population is distributed throughout the brain in both parenchymal and extraparenchymal spaces. Furthermore, this population is transcriptionally distinct and exhibits a transcriptional signature consistent with the T_RM_ observed in acute viral infections. Finally, we establish that the CD103^+^ T_RM_ population has an intrinsic capacity to produce both IFN-γ and TNF-α, cytokines critical for parasite control within the central nervous system (CNS). The contribution of this population to pro-inflammatory cytokine production suggests an important role for T_RM_ in protective and ongoing immune responses in the infected CNS.

**Accession number:** **GSE95105**

## Introduction

Persistent infections in the brain present an especially daunting challenge for the immune response due to the unique set of rules governing entry of peripheral cells and molecules into this tissue (1). Immune surveillance of the brain occurs entirely in the cerebrospinal fluid, and T cell infiltration into the parenchyma occurs only during injury and inflammation (2–4). During chronic infection with the intracellular protozoan parasite *Toxoplasma gondii*, there is a well-regulated protective response within the brain mediated by infiltrating T cells, which are required to prevent fatal reactivation (5). This continuous recruitment and the presence of activated CD4^+^ and CD8^+^ T cells in the central nervous system (CNS) represent the generation of protective immunity for the host, but fail to clear the parasite, which continues to reside as cysts within neurons (5, 6). The basis and composition of the long-term protective T cell response in the brain remains an active area of research.

Although protection could be mediated in part by a continuous pool of effector cells, there is also evidence of the involvement of memory T cell subsets (1, 7–9). Mice chronically infected with *T. gondii* exhibit protection against challenge with the virulent RH strain of the parasite, which is lethal in naïve mice (10). Persisting antigen is not required for such protection as infection of mice with attenuated strains of *T. gondii* confers protection upon rechallenge (10, 11). This suggests that the memory populations generated during chronic *T. gondii* infection are indeed functional, but it is unclear whether there is a distinction in the protection afforded by effector and memory T cell subsets in an environment of persistent antigen. Several subsets of memory T cells have been established, including central memory, effector memory, and tissue-resident memory (T_RM_) cells (12, 13). During chronic infection, memory T cells require unique survival signals (14) and can acquire distinct phenotypes, including an exhausted/attenuated phenotype (15). In chronic *T. gondii* infection, the recent discovery of a T cell population in an intermediate state (T_INT_) between memory and effector status provides an important clue to understanding the coordination of the T cell response in this context (9). Nevertheless, during chronic infection, the unique role for a memory response as opposed to the effector response remains undefined.

The location of the parasite in the parenchyma of the brain offers a potential role for T_RM_ cells in protection against parasite reactivation. T_RM_ cells have been implicated in the recruitment of peripheral lymphocytes and dendritic cell activation/maturation *via* secretion of pro-inflammatory cytokines and chemokines (16, 17). The T_RM_ population is characterized by expression of the activation marker CD69, which in tandem with the suppression of the tissue egress axis KLF2/S1PR1, ensures T_RM_ do not recirculate and remain localized in the tissue. Although not expressed by all T_RM_, the expression of the integrin CD103 is a defining marker of tissue residency. Typically, CD103 tethers T_RM_ to epithelial tissues through binding to its ligand E-cadherin (18, 19). This positions these cells optimally for a sensing and alarm function at the site of infection (16, 17, 20, 21), suggesting that this memory T cell subset is critical for a first-line protective response to localized infection. Much of the work on T_RM_ has been accomplished by studying acute infection models (22–26) where infection is resolved and antigen is cleared. This includes viral infection in the CNS (23) and parasitic challenge in the skin and liver (27, 28). Indeed, memory is frequently defined as persistent cells in the absence of infection. Yet during *Toxoplasma* infection, we observed a significant population of CD103^+^ cells in the brain. This provoked the question of whether the expression of CD103 defined a T_RM_ population during chronic infection of the CNS or whether it represented transient expression by a more common effector population.

Here, we show that a population with a T_RM_ phenotype (CD8^+^ CD69^+^ CD103^+^) exists in the brain during the chronic stage of infection, and such a population is not confined to endothelial tissues but is observed throughout the brain. In our model, expression of CD103 defines a transcriptionally distinct population that is consistent with the established literature on T_RM_ (23, 25). Furthermore, this population has a significantly greater capacity to produce the pro-inflammatory cytokines TNF-α and IFN-γ. Thus, even in the context of continuous antigen exposure, recruitment, and exhaustion of effector cells, there exists a population of CD8^+^ CD103^+^ T cells that exhibit a transcriptional profile characteristic of T_RM_. Their generation alone is not clearly sufficient to eliminate a chronic parasitic infection from the brain but may be critical nevertheless for host protective immunity.

Therefore, the presence of a population of T_RM_ during *T. gondii* infection is relevant not only to the immune response against the parasite at this stage but also to more fundamental questions regarding the role of T_RM_ and other memory subsets during chronic infection where significant antigen persists.

## Materials and Methods

### Mice and Parasites

Two type II strains of *Toxoplasma* were used to allow the quantification of parasite-specific T cells and to maximize the ability to see cysts in the brain. First, a strain engineered to secrete ovalbumin (Pru-OVA) (29) was maintained *in vitro* in human foreskin fibroblasts (HFF) grown in complete DMEM (90% DMEM, 10% fetal bovine serum, 1% penicillin/streptomycin). After infecting HFFs, parasites were grown in D10 media (70% DMEM, 20% M199, 10% fetal bovine serum, 5% penicillin/streptomycin, 5% gentamycin) with chloramphenicol. Parasites were purified for infection by passage through a 22.5-gauge needle followed by passage through a 5.0-mm nylon filter. After centrifugation at ~2,000 × *g* for 10 min at 4°C, parasites were counted and resuspended in an appropriate volume of 1× PBS. Ten thousand tachyzoites were intraperitoneally (*i.p*.) injected in 200 µL PBS. Second, the Me49 strain of parasites was maintained in CBA mice. For infections, brains were harvested from chronically infected mice and homogenized in 3 mL of PBS by needle passage. After counting, cysts were resuspended in an appropriate volume of PBS to infect *i.p*. at 20 cysts per mouse in a 200 µL volume. C57Bl/6 and CBA mice were obtained from the Jackson Laboratory (Jackson ImmunoResearch Laboratories, Inc., West Grove, PA, USA). Mice were maintained in a pathogen-free environment under IACUC established protocols at the University of California, Riverside.

### Flow Cytometry

Before harvest, mice were intracardially perfused with 20 mL ice-cold PBS, with perfusion confirmed by the white appearance of the brain and lack of red blood cells during tissue processing. Mononuclear cells were isolated from the brain by mincing and subsequent homogenization *via* passage through an 18-gauge needle in complete RPMI (86% RPMI, 10% FBS, 1% penicillin/streptomycin, 1% l-glutamine, 1% NEAA, 1% sodium pyruvate <0.01% β-mercaptoethanol). The resulting suspension was incubated at 37°C with 3 mg DNAse and 100 µg collagenase for 1 h and 45 min. After incubation, the suspension was passed through a 70-µm strainer, and mononuclear cells were isolated using a density gradient spun at 2,000 rpm for 25 min with no brakes. The density gradient consisted of a 60% Percoll solution in cRPMI overlayed with a 30% Percoll solution in PBS. Brain mononuclear cells (BMNCs) were isolated from the interphase, counted and washed in FACS buffer, blocked for 10 min in F_c_ block (BD Biosciences), and then incubated with a panel of antibodies against CD3, CD8, CD4, CD69, and CD103 (eBioscience) for 30 min protected from light. Samples were then washed and fixed in 4% PFA. Dextramer staining was performed as follows: Prior to surface staining, samples were incubated with ovalbumin-specific (SIINKFEKL) MHC I dextramer (Immudex) at room temperature for 45 min, protected from light. The finished samples were resuspended in FACS buffer and analyzed using a FACS Canto from BD Biosciences. Flow cytometry analyses were performed using FlowJo 10.1, and statistical analyses were performed using Prism 6.

For sorting of cell populations for RNA-Seq, BMNCs were isolated as described above. Splenocytes were isolated as follows: spleens were homogenized in a 40-µm strainer using the blunt end of a 3-mL syringe. The homogenate was washed with cRPMI, and red blood cells were lysed using ACK buffer from Lonza. Cells were then washed and counted. CD8^+^ T cells were isolated from spleen and BMNCs using negative selection columns from R&D systems. The purified CD8^+^ T cells were then incubated with anti-CD103 antibodies according to the protocol described above. Cells were suspended at a concentration of 3 × 10^6^ cells/mL and then sorted using a FACS Aria from BD Biosciences. Cell sorting was performed at the Institute for Integrative Genome Biology at UC Riverside.

### *In Situ* Immunofluorescent Staining

Immediately following excision, sagitally bisected brain tissue was flash-frozen in a bath of isopentane cooled with dry ice. Frozen organs were then put into a standard Tissue-Tek cryomold, filled with optimal cutting temperature solution (also from Tissue-Tek), put on dry ice, and subsequently stored at −80°C. Serial sections of 12 µm were prepared on a standard Cryostat machine (LEICA/CM1850). For *in situ* immunofluorescent staining, frozen tissue sections were fixed in 75% acetone/25% ethanol and then blocked for 10 min in 10% donkey serum prior to staining. For the primary antibody incubation, conjugated anti-mouse CD8 and CD103 were added to three panels containing anti-mouse biotinylated E-cadherin, anti-mouse laminin, or anti-*T. gondii*. Antibodies against CD8, CD103, and E-cadherin were from eBioscience, antibodies against laminin from Cedarlane Laboratories, and anti-*Toxoplasma* from Abcam. The sections were incubated for 3 h at room temperature. After washing, the sections were incubated for 1 h at room temperature with appropriate secondary antibodies (streptavidin for anti E-cadherin). Samples were mounted in ProLong Gold with DAPI (Invitrogen) for nuclear counterstaining. Images were collected on either a Leica DMI 6000B epifluorescent or a Leica SP5 scanning confocal microscope (Leica Optics), and data were analyzed and quantified using Volocity 6.1 (Perkin-Elmer).

### RNA-Seq Analysis

To obtain sufficient RNA for analysis, CD8 T cells were isolated from pooled leukocytes of brain and spleen of at least *n* = 5 mice. This was repeated to obtain four trials to serve as biological replicates in analysis. The isolated CD8 T cells were sorted according to expression of CD103, and RNA was extracted from sorted groups using an RNeasy Mini Kit from Qiagen. Multiplexed cDNA libraries were generated, which included brain CD103^+^, brain CD103^−^, spleen CD103^+^, and spleen CD103^−^ CD8 T cells. Of the four trials, libraries of sufficient quality for sequencing could not be generated for two brain CD103^+^ samples and one brain CD103^−^ sample. RNA concentration and quality were analyzed by running an RNA Nano chip on an Agilent 2100 Bioanalyzer. mRNA was enriched using a RiboGone Mammalian kit from Clontech. cDNA libraries were generated using a SMARTer stranded RNA-Seq kit, also from Clontech. Concentration and quality of libraries were measured with a High Sensitivity DNA chip (Agilent). The resulting libraries were multiplexed, and single-end 50 bp sequencing was performed at the UC Riverside Genomics Core facility using the Illumina HiSeq 2500. After demultiplexing and QC of the resulting FASTQ files, alignment and differential gene expression analysis were performed with the s*ystemPipeR* workflow^1^ (30). Quality control and trimming were conducted using FastQC^2^ and Trim Galore,^3^ respectively. The reads were then aligned to the mouse genome using Tophat2 (31), and transcripts per gene were counted using *GenomicRanges* package in R (32). Differential gene expression analysis was performed following TMM normalization using *edgeR* (30, 33). MetaCore software (Thomson Reuters) was used for functional enrichment analysis. To conduct functional enrichment analysis, only DEGs with a fold change (FC) >2 and FDR <5% were used. We further excluded genes with very low counts by only selecting gene with a mean reads per kilobase of transcript per million mapped reads (RPKM) >1. RPKM served as a normalized value for read count and was obtained *via GenomicRanges*.

### Microarray Analysis

Microarray data from the studies by Wakim et al. and MacKay et al. were obtained from the NCBI Gene Expression Omnibus database under accession number GSE39152 (25) and GSE47045 (26). Differential gene expression analysis was conducted using GEO2R. Genes with a corrected *p* < 0.05 were used for subsequent analyses, including functional enrichment analysis. For the Wakim et al.’s data, differential gene expression analysis was conducted between brain CD8^+^ CD103^+^ (*n* = 5) and brain CD8^+^ CD103^−^ (*n* = 3) samples. For MacKay et al. data, differential gene expression analysis was conducted between skin T_RM_ (*n* = 3) and spleen T_CM_ plus spleen T_EM_ (*n* = 6) samples.

### Restimulation Assay

Brain mononuclear cells were collected from mice by 5 weeks postinfection and incubated for 6 h in complete T cell media (86% DMEM, 10% FBS, 1% penicillin/streptomycin, 1% l-glutamine, 1% NEAA, 1% sodium pyruvate, <0.01% β-mercaptoethanol) with 10 µg/mL BFA. Cells were treated with 10 µg/mL αCD3/αCD28 (BD Biosciences) antibody or media alone. After restimulation, cells were collected, and surface staining was performed as described above. Intracellular cytokine staining was performed with the FoxP3/transcription factor staining buffer kit from eBioscience. Antibodies against IFN-γ and TNF-α were obtained from eBioscience. Flow cytometry analyses were performed using FlowJo 10.1, and statistical analyses were performed using Prism 6.

## Results

### Kinetics and Distribution of CD103^+^ CD8 T Cells in the *Toxoplasma*-Infected Brain

**Figure 1:**
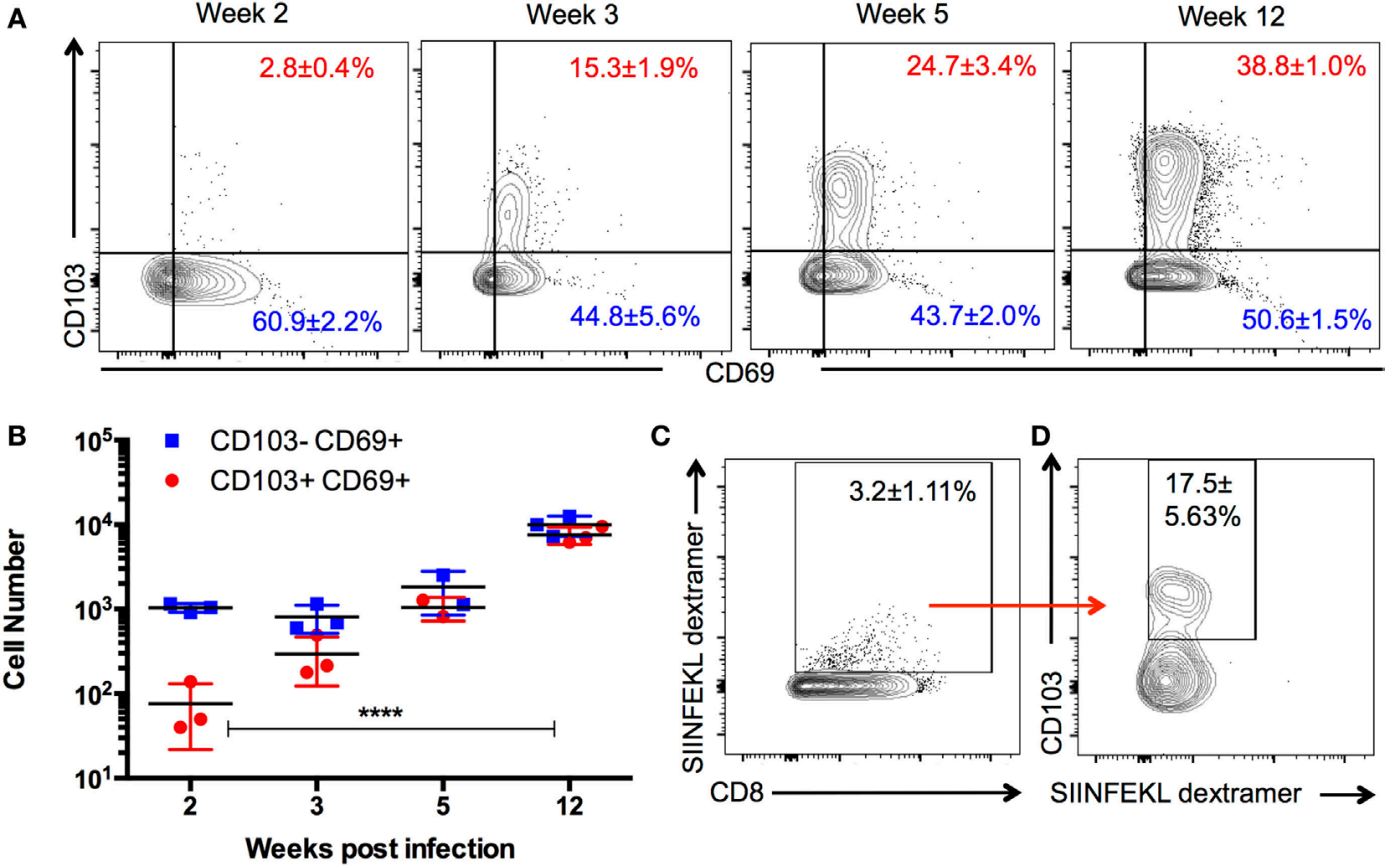
**Kinetics and specificity of CD103^+^ CD8 T cells in the *Toxoplasma*-infected brain**. Brain mononuclear cells harvested from *Toxoplasma gondii*-infected mice at indicated time points and gated on live CD3^+^ CD8^+^ cells. **(A)** Numbers indicate the proportion of CD69^+^ CD103^+^ and CD69^+^ CD103^−^ CD8 T cells in the brain at the indicated time points. **(B)** Absolute cell counts for the data are shown in panel **(A)**. **(C)** Percentage of dextramer^+^ CD8 T cells in the brain. **(D)** Percentage of SIINFEKL dextramer^+^ CD8 T cells expressing CD103. Data from week 12 postinfection. Numbers indicate average ± SEM of three biological replicates for each time point. Significance was determined using two-way ANOVA with multiple comparisons post test. ****p < 0.0001. Data are representative of two independent experiments with similar results.

T_RM_ are defined as a persisting population of T cells that remain localized to the tissue, that is, non-recirculating. To determine whether such a population exists in the brain during chronic *T. gondii* infection, we investigated whether a stable T_RM_ phenotype could be observed across the course of infection and whether such a phenotype would localize to the parenchyma. Therefore, we enumerated the distribution and kinetics of CD8 T cells in the brain expressing the residency markers CD103 and CD69. There are no T cells in a naïve uninfected brain (1, 34); however, by 2 weeks postinfection, representing the late acute stage, there is a small proportion (2 ± 0.4%) of CD8 T cells exhibiting a CD103^+^ CD69^+^ phenotype. This population significantly increases (*p* < 0.0001) over the course of infection, and at the late chronic stage (12 weeks postinfection), 38 ± 1% of CD8^+^ T cells were observed to be positive for CD103 and CD69 (Figures 1A,B). Thus, by the late stages of chronic infection, this phenotype forms a substantial percentage of the CD8 subset in the brain relative to all other phenotypes contained in the CD103^−^ group. In the periphery, CD103^+^ cells are observed to accumulate in the spleen and lymph node over time; however, such cells did not express CD69 in significant proportions (Figure S1 in Supplementary Material) and are more transcriptionally consistent with a quiescent/naïve population (Figure S2 in Supplementary Material) (35). In contrast, the brain has no population of CD103^+^ CD8 T cells that do not express CD69 (Figure 1A), and therefore, the use of this marker for subsequent analysis was redundant.

Previous work has suggested that the majority of T cells in the brain are specific for the parasite, and there is little evidence of a bystander population (36). However, to rule out the possibility that CD103-expressing cells are not directly responding to parasite infection, we used parasites engineered to express ovalbumin along with ovalbumin-specific (SIINFEKL) MHC I dextramer (29, 36) to test the antigen specificity of this population. At 12 weeks postinfection, approximately, 3% of CD8^+^ cells in the brain are bound by the SIINFEKL dextramer, a significant proportion of which express CD103 (Figure 1C). These data do not definitively establish that all CD103^+^ CD8 T cells in the brain are parasite specific nor does it indicate the full repertoire of parasite antigens to which CD103^+^ CD8 T cells could respond. Nevertheless, it does support the view that brain CD103^+^ CD8 T cells are not simply a bystander population and is consistent with the previous work demonstrating that retention of T cell populations in the brain requires cognate antigen at this site (Figure S7 in Supplementary Material) (36, 37).

**Figure 2:**
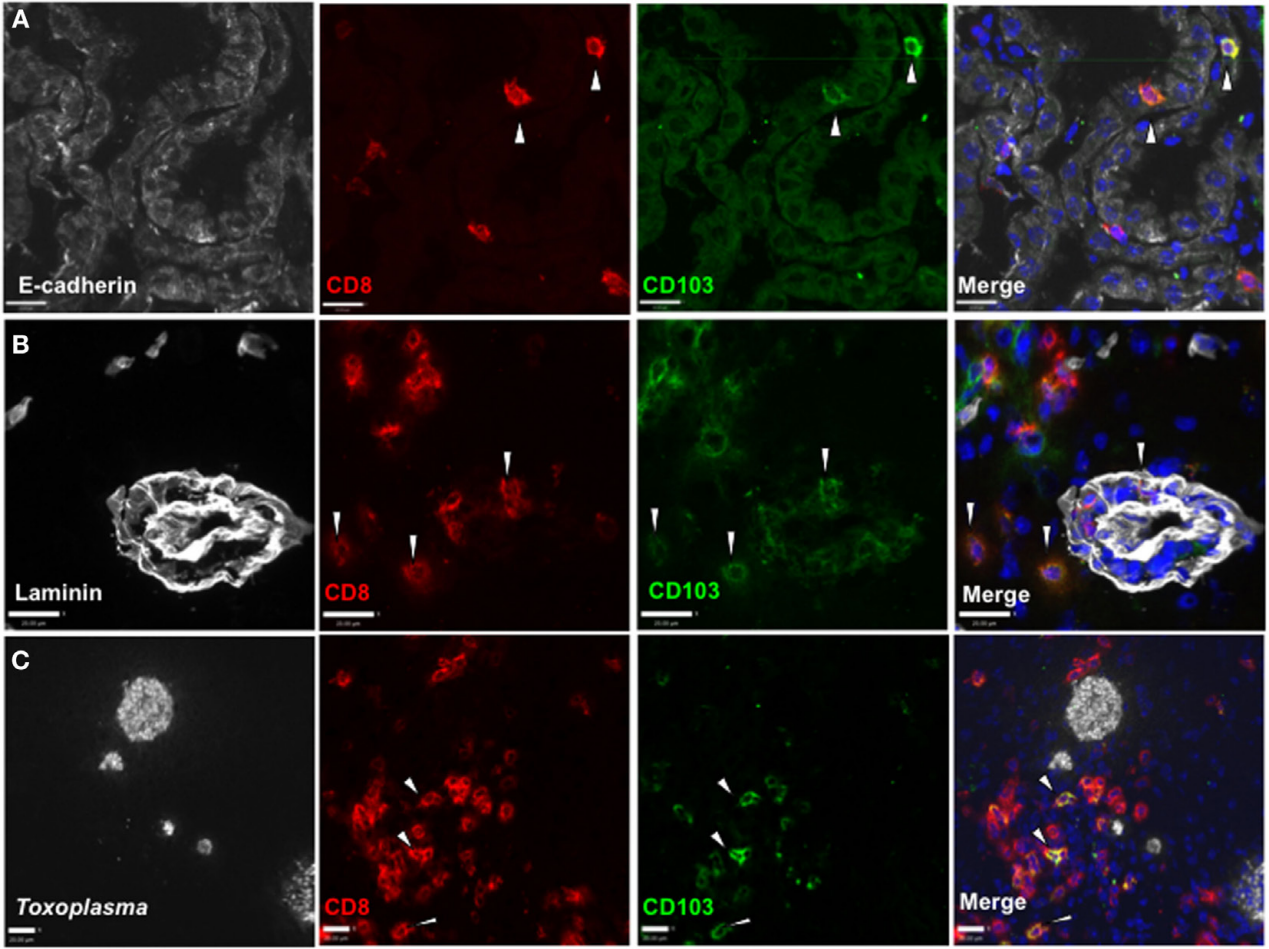
**Distribution of CD103^+^ CD8 T cells in the *Toxoplasma gondii*-infected brain**. Immunofluorescent staining *in situ* for CD8 and CD103 in representative sagittal section-infected brain at 5 weeks postinfection. **(A)** E-cadherin staining in choroid plexus of lateral ventricle, 40× magnification, zoomed in. **(B)** Laminin staining for vasculature of frontal cortex, 40× magnification, zoomed in. **(C)** *T. gondii* staining in the frontal cortex, 25× magnification, zoomed in. All images are representative of *n* = 3 biological replicates at 5 weeks post infection. White arrows indicate examples of staining positive for both CD8 and CD103. Scale bar indicates 20 µm.

To determine whether CD103^+^ CD8 T cells localize to distinct environments within the brain, we used *in situ* immunofluorescent staining. Expression of CD103 by CD8 T cells is observed at entry points to the brain, including the choroid plexus, where it associates with the CD103 ligand E-cadherin (Figure 2A). In addition, this population can be found within the perivascular space (Figure 2B) and within the parenchyma, despite little expression of E-cadherin at these sites (38). Finally, this population can be observed in proximity to cysts and individual parasites (Figure 2C). Quantification of the distance of CD103^+^ cells from cysts was highly variable (80–160 µm), and there was no correlation between cyst and CD103^+^ cell number (Figure S3 in Supplementary Material).

Together these data indicate that a population of CD103^+^ CD8 T cells can be observed throughout the chronic stage and form a substantial proportion of the CD8 subset in the infected CNS. *In situ* immunofluorescent staining revealed that CD103 expression is not confined to a certain area of the brain or specifically localized to the regions of infection. Our observation that CD103^+^ CD8 T cells are present within the perivascular space suggests that this phenotype may be acquired before entry into the parenchyma. Together, these data raised the possibility that CD103 did not define a homogenous population of T cells within the brain but rather a functionally diverse set of cells having in common expression of CD103.

### CD103^+^ CD8 T Cells Exhibit a Distinct Transcriptional Profile Relative to Other CD8 T Cells in the Brain and Periphery

The heterogeneity of infiltrating T cells during chronic infection (8, 36, 39, 40) required we exclude the possibility of CD103 expression masking a group of functionally and phenotypically diverse cells that are not T_RM_. Furthermore, expression of CD103 is not the limiting phenotype for T_RM_, that is, a memory T cell can be resident (non-recirculating) without expressing CD103 (13). To address whether CD103 defines a transcriptionally distinct population within the brain that is uniquely characteristic of T_RM_, RNA-Seq analysis was performed on sorted cells from infected mice at 4 weeks postinfection.

**Figure 3:**
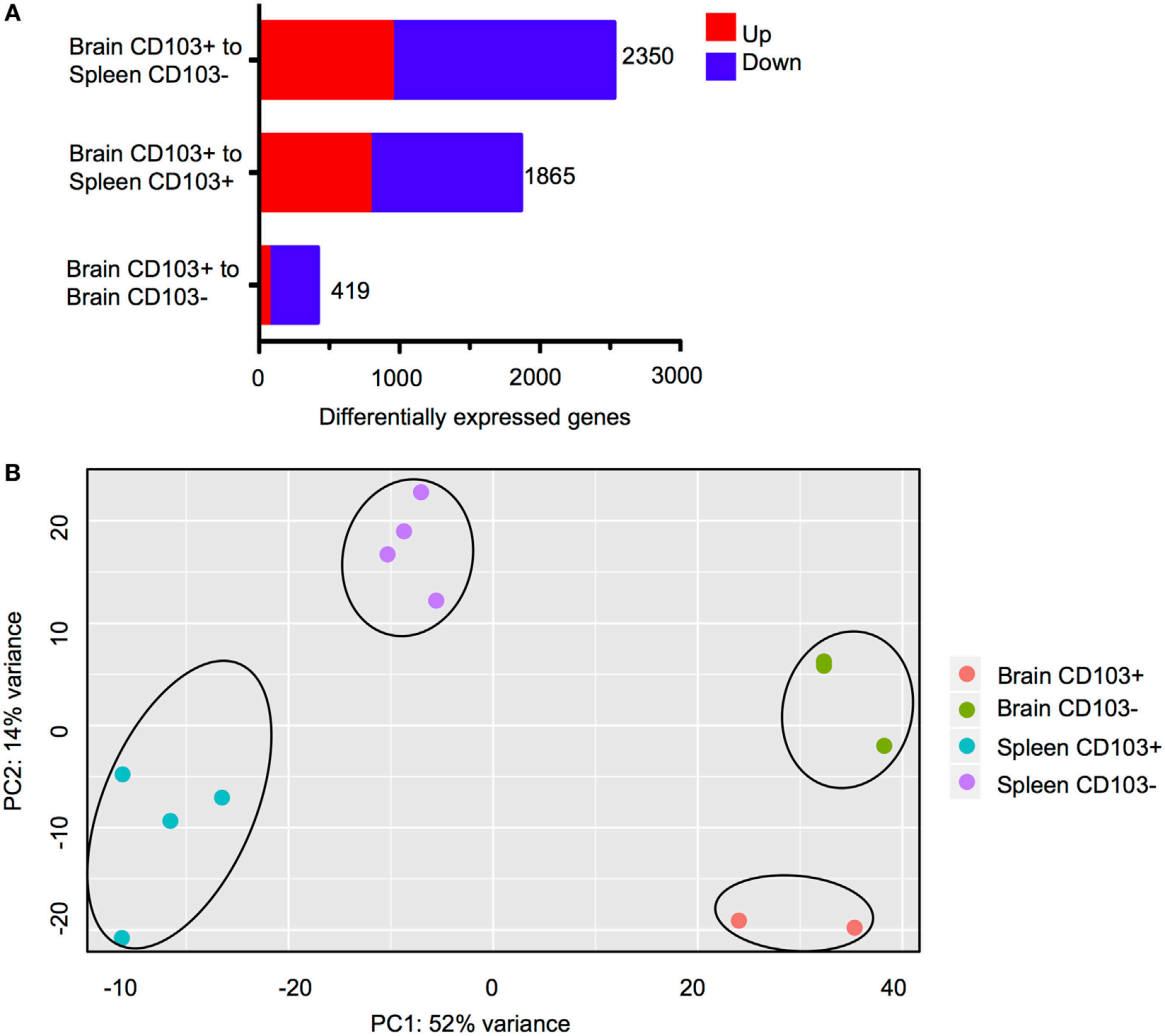
**Transcriptional characteristics of brain CD103^+^ CD8 T cells**. CD8 T cells were isolated from the brain and spleen of chronically infected mice and sorted according to CD103 expression. **(A)** Differentially expressed genes (DEGs) for the three indicated analyses. Numbers indicate combined upregulated and downregulated DEGs with fold change >2 and FDR <5%. **(B)** PCA plot for normalized read counts (reads per kilobase of transcript per million mapped reads) for all samples.

Three comparisons between CD8^+^ subsets were carried out (Figure 3A). The first comparison, brain CD103^+^ to brain CD103^−^, specified whether CD103 defined a transcriptionally distinct population within the brain. The second comparison, brain CD103^+^ to spleen CD103^+^, specified whether CD103 defined a transcriptionally distinct CD8 T cell population when compared to a putatively naïve peripheral population of the same phenotype. Finally, the brain CD103^+^ to spleen CD103^−^ comparison specified whether CD103 defined a transcriptionally distinct population of CD8 T cells when compared to a putatively activated peripheral population. The comparison of brain CD103^+^ to spleen CD103^−^ yielded the greatest number of DEGs (2,350) (Figure 3A). This provided preliminary evidence that the brain CD103^+^ CD8 T cell group is transcriptionally dissimilar to both local and peripheral CD8 T cells. To confirm these data, principal component analysis was performed to determine the variance in RPKM across all groups (Figure 3B). The resulting plots demonstrate that the brain CD103^+^, brain CD103^−^, spleen CD103^+^, and spleen CD103^−^ populations are transcriptionally distinct from each other. PC1 appears to define the organ, spleen versus brain, whereas PC2 appears to define CD103 expression. PC1 had a loading of 12.90, and PC2 had a loading of 14.14. The most variable gene in PC1 was transthyretin, whereas the most variable gene in PC2 was *S1pr5* (Figure S4 in Supplementary Material). These data lend support to CD103 expression defining a distinct and homogenous population of CD8 T cells within the chronically infected brain.

### CD103^+^ CD8 T Cells Exhibit a Distinct Transcriptional Profile Relative to Brain CD103^−^ CD8 T Cells that Is Consistent with T_RM_

**Figure 4:**
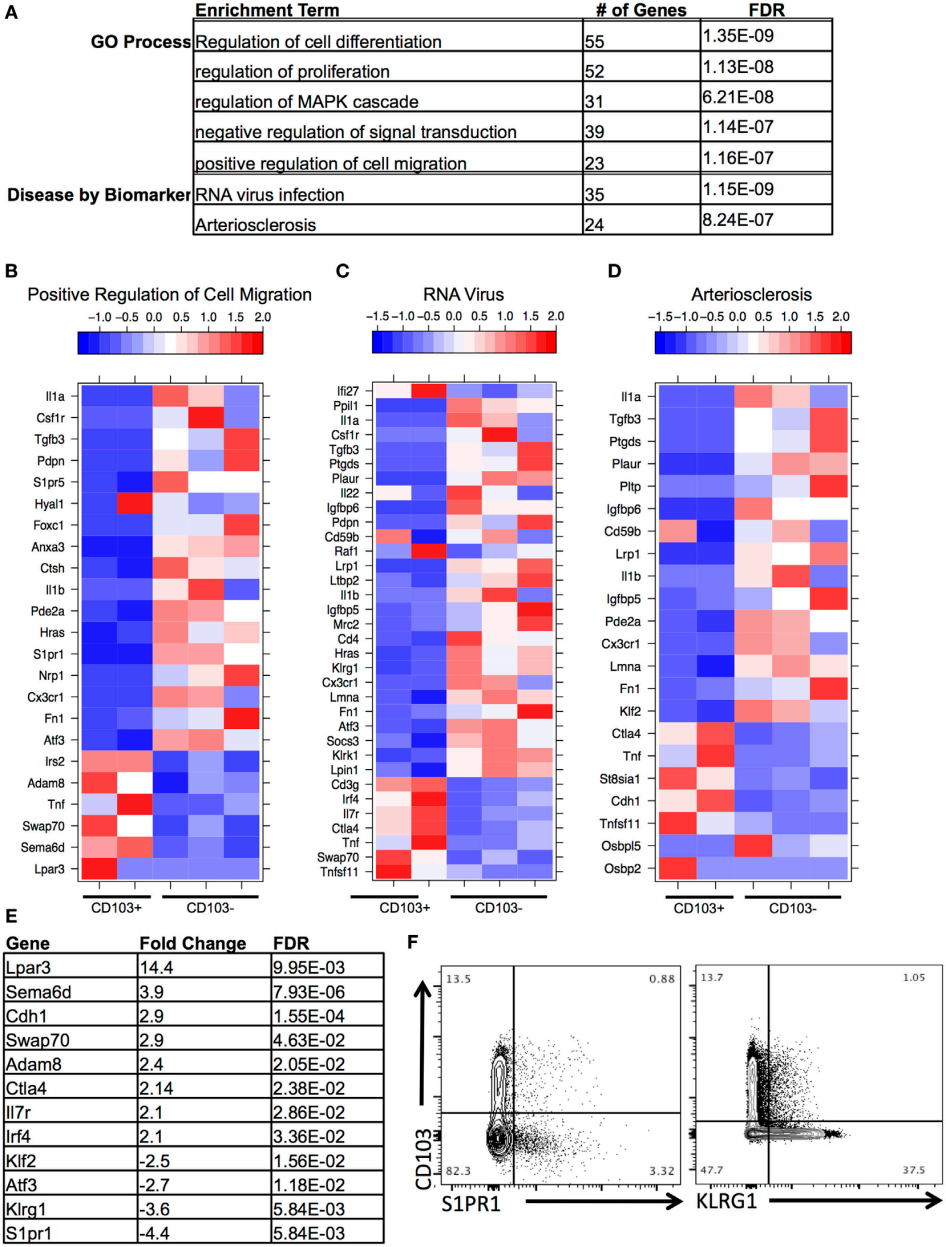
**Distinct transcriptional profile of brain CD103^+^ CD8 T cells relative to brain CD103^−^ CD8 T cells**. **(A)** After conducting differential gene expression analysis between brain CD103^+^ and brain CD103^−^ CD8 T cells, the genes with a fold change >2, FDR <5%, and mean RPKM >1 were input into MetaCore for functional enrichment. Of the 239 genes input, the following terms with FDR <5% were extracted from the categories “Disease by Biomarker” and “GO Process.” **(B)** Heatmap of genes under GO process term “Positive regulation of cell migration.” **(C)** Heatmap of genes under Disease term “RNA virus.” **(D)** Heatmap of genes under enrichment term “Arteriosclerosis.” Individual replicates in heatmap are pooled from *n* = 5 mice. Values in legend are scaled values representative of RPKM. Red indicates a highly expressed gene, and blue indicates a gene with a low expression value. **(E)** Table of fold changes and false discovery rate for genes relevant to tissue residence and memory phenotype. **(F)** Flow cytometry for differential expression of S1PR1 and KLRG1.

Next, DEGs were analyzed between CD103^+^ CD8 T cells and CD103^−^ CD8 T cells within the brain. Functional enrichment analysis revealed that the DEGs enriched to GO terms such as cell migration, proliferation, and differentiation (Figures 4A,B). These processes are critical aspects of T cell response to infection, T cell signaling, and activation. In addition, these DEGs had significant overrepresentation of notable disease terms—“RNA virus infection” (Figures 4A,C) and “arteriosclerosis” (Figures 4A,D). In support of the view that this is a memory population, the terminal differentiation marker *Klrg1*, expression of which defines terminally differentiated effector cells, is downregulated in the brain CD103^+^ population (FC = −3.5), whereas *Il-7r*, a receptor that defines memory cells is upregulated (FC = 2.1) (Figures 4C,E). Furthermore, consistent with tissue residency is the downregulation of *Klf2* (FC = −2.5) and *S1pr1* (FC = −4.4) in the brain CD103^+^ population (Figures 4B–E), inhibiting recirculation of cells back to lymphoid organs (Schenkel and Masopust). To validate differential expression, flow cytometry of BMNCs was conducted. This confirms downregulation of both S1PR1 and KLRG1 at the protein level in CD103^+^ CD8^+^ T cells (Figure 4F). The percentage of CD103^−^ S1PR1^+^ CD8 T cells is approximately threefold higher that of the CD103^+^ S1PR1^+^ subset (0.88 versus 3.32%) (Figure 4F). A total of 37.50% of CD103^−^ CD8 T cells were KLRG1^+^, whereas 1.05% of CD103^+^ CD8 T cells were KLRG1^+^ (Figure 4F).

Additional changes of interest in the brain CD103^+^ population included upregulation of the co-inhibitory receptor *Ctla4* (FC = 2.14) and the transcription factor *Irf4* (FC = 2.1) and downregulation of the transcription factor *Atf3* (FC = −2.6) (Figures 4C–E). *Ctla4* is among the first co-inhibitory receptors to be expressed and could provide evidence of increased antigen engagement in this subset (41). Although several co-inhibitory receptors were upregulated in the brain CD103^+^ group compared to the spleen CD103^+^ group, including *Pdcd1* (FC = 77.7), *Lag3* (FC = 9.2), and *Tigit* (FC = 27.7) (not shown), only *Ctla4* was uniquely upregulated relative to other CD8 T cells in the brain.

A set of genes associated with extracellular adhesion and migration were upregulated in the brain CD103^+^ population, including *Cdh1* (E-cadherin, FC = 2.9), *Adam8* (FC = 2.3), *Swap70* (FC = 2.8), *Sema6d* (FC = 3.7), and *Lpar3* (FC = 13.9) (Figures 4B,E). Excluding *Sema6d*, these are novel upregulated genes in T cells (42–45). Both *Sema6d* and the E-cadherin gene were also upregulated relative to peripheral spleen CD8 T cells (both CD103^−^ and CD103^+^), supporting the possibility that these genes are unique determinants of residency for the brain CD103^+^ population in our model (Figure S5 in Supplementary Material). This was also the case for the downregulated genes *S1pr1* and *Klf2* (Figure S5 in Supplementary Material).

### T_RM_ from the *Toxoplasma*-Infected Brain Share a Core Set of Differentially Expressed Genes with T_RM_ from Vesicular Stomatitis Virus (VSV)

**Figure 5:**
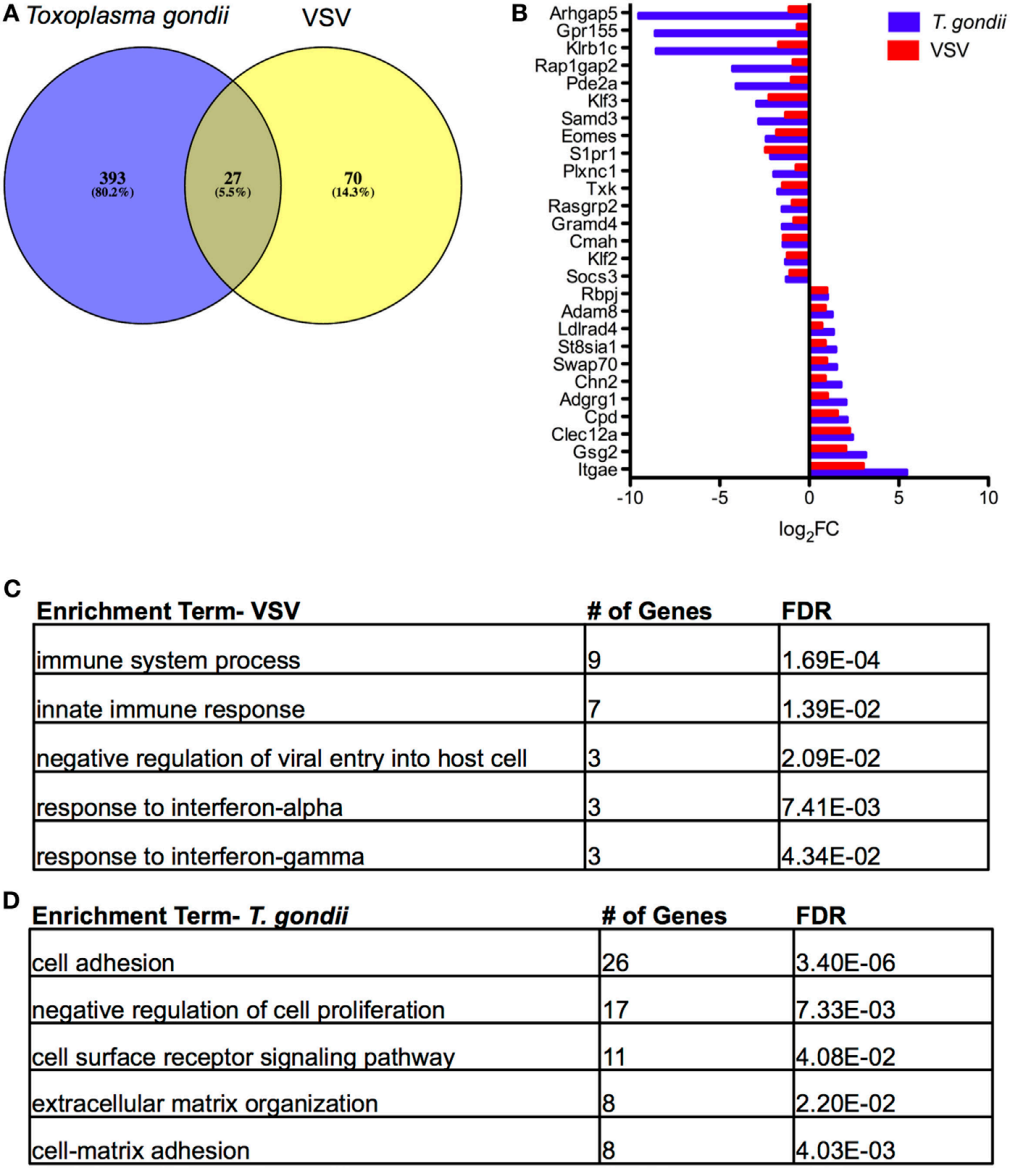
**Comparison of tissue resident memory T cells (T_RM_) from acute vesicular stomatitis virus (VSV) to *Toxoplasma gondii***. Microarray data from the study by Wakim et al. were obtained and analyzed *via* GEO. **(A)** Venn diagram of DEGs in *T. gondii* and VSV (25) for the brain CD103^+^ CD8 T cells relative to brain CD103^−^ CD8 T cells. **(B)** Comparison of fold changes for the 27 differentially expressed genes common to both models. RNA-Seq DEGs were determined according to the following criteria: fold change >2, FDR <5%, *p* < 0.05, and mean RPKM >1. Microarray DEGs were significant if the *p* value and adjusted *p* value were less than 5%. **(C)** Results of enrichment analysis for DEGs unique to microarray analysis of T_RM_ in VSV. **(D)** Results of enrichment analysis for DEGs unique to RNA-Seq analysis of T_RM_ in *T. gondii* infection.

The original studies of T_RM_ in the brain by Wakim and colleagues included microarray analysis to generate a transcriptional profile of T_RM_ in a VSV model of acute and resolved brain infection (25). We compared DEGs from this model with the RNA-Seq data generated in our chronic model, focusing specifically on brain CD103^+^ CD8 T cells relative to brain CD103^−^ CD8 T cells. The results of our Venn analysis reveal a set of 27 genes (~5%) common to both transcriptional profiles (Figure 5A). *S1pr1, Klf2*, and *Adam8* are contained within this set, suggesting similar mechanisms of residency between the two models (Figure 5B). *Swap70* and *Eomes* are also represented in this group. A remaining 70 genes are unique to VSV, and a remaining 393 genes are unique to *T. gondii*. The top enrichment terms for these sets of genes were “immune system process” and “cell adhesion” (Figures 5C,D, respectively). When our data were compared to data from skin T_RM_ generated from HSV infection (26), we observed a similar set of 33 differentially expressed genes, comprising 4.1% of the total DEGs (Figure S6 in Supplementary Material). Although the majority of differentially expressed genes in each model were unique to the model, a core profile of genes associated with tissue residency was common to all models (Figure 5; Figure S6 in Supplementary Material).

### CD103^+^ CD8 T Cells Produce a Significantly Greater Percentage of Pro-inflammatory Cytokines

Several cytokines and receptors including *Tgfb3, Il1a, Il1b, Il22*, and *Csf1r* are downregulated in brain CD103^+^ CD8 T cells relative to brain CD103^−^ CD8^+^ T cells (Figures 4B–D). The TGF-β receptor was upregulated (FC = 2.75) in brain CD103^+^ CD8 T cells, consistent with previous reports of the requirement for TGF-β in generation of this population (46, 47). Notably, the pro-inflammatory cytokine *Tnf* is significantly upregulated in CD103^+^ CD8 T cells (FC = 2.5 relative to brain CD103^−^) (Figures 4B–D; Figure S5 in Supplementary Material). T_RM_ are reported to produce this cytokine although their ability to do so relative to other CD8 T cell subsets has not been previously validated *via* flow cytometry (16, 25, 26).

**Figure 6:**
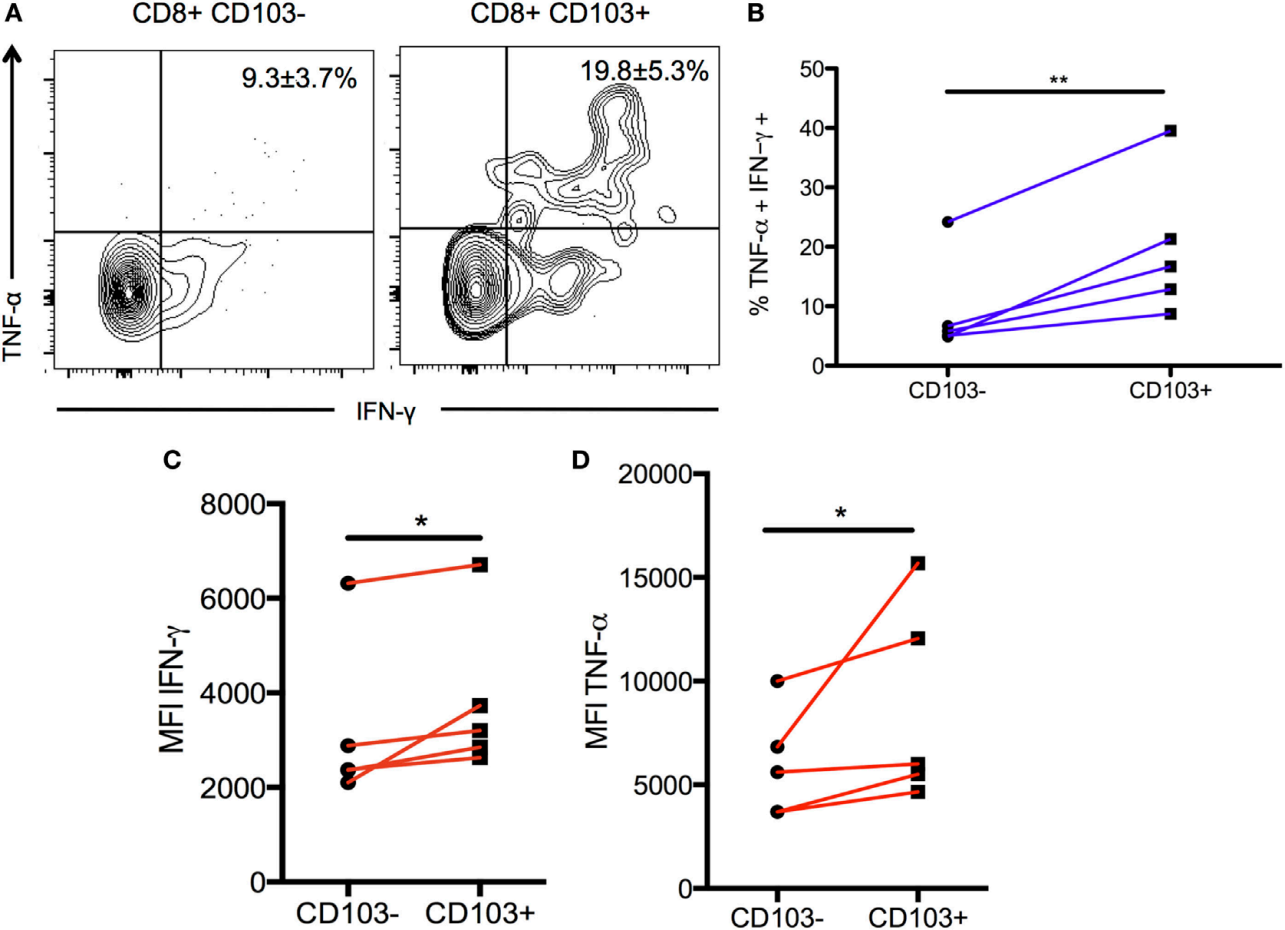
**Production of pro-inflammatory cytokines in CD103^+^ CD8 T cells**. Brain mononuclear cells from chronically infected mice were restimulated with α-CD3/α-CD28 antibodies for 6 hours and intracellular cytokine staining was performed. Data were gated on live CD3^+^ CD8 T cells and then split into CD103^+^ and CD103^–^ subsets for subsequent analysis. **(A)** Representative flow plot indicating percentage of restimulated CD103^+^ and CD103^–^ CD8 T cells producing IFN-γ and TNF-α. Numbers indicate mean percentage ± SEM of *n* = 5 biological replicates. **(B)** Paired data plot of the data shown in **(A)**. **(C)** Geometric MFI of IFN-γ production comparing CD103^+^ to CD103^–^ CD8 T cells subsets. **(D)** Geometric MFI of TNF-α production for the same comparison. Significance was determined using a paired two-sample *t*-test. *p < 0.05, **p < 0.01; *p* values are one tailed. Data are representative of two independent experiments with similar results.

To validate and confirm the increase in *Tnf* gene expression in the CD103-expressing cells, BMNCs from chronically infected mice were restimulated and production of IFN-γ and TNF-α was compared between the CD103^+^ and CD103^−^ subsets. The proportion of CD103^+^ T cells producing both IFN-γ and TNF-α was significantly greater than cells not expressing CD103 (19.6 ± 5.3 versus 9.3 ± 3.7%; *p* < 0.01) (Figures 6A,B). Furthermore, of CD103^+^ CD8 T cells expressed more total TNF-α and more total IFN-γ on a per cell basis than their CD103^−^ counterparts (for IFN-γ: 3,824 ± 787% versus 3,208 ± 746 MFI; *p* < 0.05) (for TNF-α: 8,778 ± 2,165% versus 5,966 ± 1,171 MFI; *p* < 0.05) (Figures 6C,D). Since both TNF-α and IFN-γ are critical for protection against toxoplasmic encephalitis (TE) (5, 48), these data suggest that the CD103^+^ T_RM_ population contributes to protection within the brain at the chronic stage.

## Discussion

These data support the conclusions that (1) CD103^+^ CD8 T cells in the brain during chronic *T. gondii* infection are a transcriptionally distinct population, (2) this population exhibits transcriptional signatures characteristic of T_RM_, and (3) this population has a distinct contribution to the pro-inflammatory protective response to the parasite.

Expression of CD103 can define a resident memory T cell, yet the T_RM_ phenotype is not restricted to CD103 expression, nor is CD103 expression exclusive to this subset (13, 47, 49–51). This is seen in our data with flow cytometry and immunohistochemistry data revealing expression of CD103 by CD4^+^ T cells and dendritic cells in the *Toxoplasma*-infected brain and CD8^+^ T cells (Figure 2; Figure S7 in Supplementary Material and data not shown) (52, 53). However, our data suggest that within brain, CD8^+^ T cells expression of CD103 does indeed represent a population of T_RM_ cells that are not restricted to the E-cadherin-expressing endothelial cells of the choroid plexus but are also found in parenchymal and extraparenchymal spaces, independently of cyst location (24, 38). Previously, effector T cells have been postulated to be essential for control of chronic infection, although the presence of a T_RM_ population is in keeping with the requirement to continuously keep cyst reactivation in check.

The reason for CD103 defining a resident memory population has not been addressed, and perhaps this is even less clear when brain tissue expresses very little of this integrin’s primary ligand, E-cadherin (38). The expression of *N*-cadherin is highly prevalent in the brain, and CD103 has the potential to bind to this cadherin instead (unpublished data). However, our observation that the E-cadherin gene is upregulated in the CD103^+^ population suggests that residency could be mediated by homotypic pairings within the CD103^+^ population. Alternatively, our data would support the concept that dendritic cells serve as a binding partner for CD103^+^ CD8 T cells and mediate residency *via* formation of the immunological synapse. This could occur *via* the expression of E-cadherin on brain-infiltrating dendritic cells (54) or, given that our brain T_RM_ upregulate *Sema6d, via* binding to plexins on dendritic cells (43, 44). Independent of cell–cell interactions, CD103^+^ cells also upregulate *Adam8*. This gene encodes for a metalloprotease implicated in cell-matrix adhesion in human PBMCs and may serve as an additional residency determinant in the brain T_RM_ population (43). In addition, this gene could play a role in facilitating cytokine shedding (55) and thus provide a mechanistic basis for the increased cytokine production in this population.

TGF-β-dependent upregulation of CD103 plays a crucial co-stimulatory role in facilitating TCR-mediated cytokine secretion for tumor specific CD8^+^ T cells (47). The role of TGF-β in the generation and maintenance of CD103^+^ T_RM_ has been previously reported (24). Our transcriptional data suggest that CD103^−^ CD8^+^ T cells may serve as a source of TGF-β, and this is strengthened by the concurrent upregulation of *Atf3* in this population (56).

Consistent reports show a dependence on dendritic cell interactions for the priming of or the retention of T_RM_, and our data are supportive of this (51, 57). This may extend to other models and tissues, particularly in the context of chronic infections. T_RM_ are observed to accumulate in cases of non-specific inflammation, but during latent viral infections, there is speculation that survival of CD103^+^ CD8 T cell populations within neural tissues is uniquely dependent on antigen presentation (24, 26, 46). A direct comparison of acute and chronic mucosal LCMV infection indicates that chronic viral infection can promote the accumulation of CD4^+^ T_RM_ in non-lymphoid tissues and upregulation of non-lymphoid tissue homing markers such as CXCR4 (58). This is in agreement with our data demonstrating the accumulation of T_RM_ in the brain over the course of infection. Finally, in a vaccinia virus model of acute skin infection, cognate antigen is not required for recruitment of CD8^+^ T_RM_ *per se*, but the local antigen profile influences the degree of accumulation and TCR repertoire of such T_RM_ (59). Taken together, these and our data support local antigen presentation as a cue for long-term maintenance and accumulation of T_RM_.

In addition to genes involved in residency determination, we also observed differentially expressed genes that suggest mechanisms of motility within the brain for CD103^+^ CD8 T cells. These include the GPCR *Lpar3* and the guanidine exchange factor *Swap70*, which are upregulated (42, 45). *Swap70* expression, although typically associated with B cells, has also been shown to initiate membrane ruffling in fibroblasts (42). Although *Lpar3* expression has not been reported in T cells, it has been reported to mediate motility and proliferation in cancer cells and therefore could play an analogous role in tissue-resident T cells (45).

Although the transcriptional profile generated during *T. gondii* infection is distinct to the transcriptional profile generated during VSV, the differentially expressed genes shared between the two profiles are consistent with a T_RM_ population (25) and suggest that a T_RM_ subset also exists during chronic infection in the CNS. Our study also demonstrates an important role for these cells in producing effector cytokines. Stimulation of CD103^+^ T cells compared to CD103^−^ led to an increase in IFN-γ and TNF-α production. Although T_RM_ have been reported to produce TNF-α, our results suggest that they have a greater capacity to produce this cytokine, compared to non-CD103^+^ T cells in the brain (16, 25). TNF-α is crucial to prevent progression of TE (48, 60, 61). Mice treated with anti-TNF-α antibodies succumb to primary infection (48), and mice lacking TNF-α receptors exhibit necrosis of brain tissue due to parasite reactivation (60, 61). More recently, a study enriching T_RM_ in the gut through depletion of circulating T cells demonstrate a critical function of TNF-α in the recruitment and activation of dendritic cells (16). This could serve as an alternate/additional mechanism by which T_RM_ are able to participate in the protective response to infection *via* production of TNF-α and recruitment of innate and/or antigen-presenting cells. Although the contribution of this subset to overall TNF-α production in the BMNC compartment is small, the significantly increased capacity to produce this cytokine relative to other CD8 T cells suggests that it may be optimized to respond in a robust fashion to inflammatory microenvironments within the brain.

The increased production of IFN-γ in brain CD103^+^ CD8 T cells relative to other CD8 T cells in the brain also points to a role for T_RM_ in protection against TE. This stimulation occurred by 5 weeks of infection. At this point, peak recruitment of effector cells has passed, and there is now a considerable population of PD-1^hi^ exhausted effector cells that have a reduced capacity for IFN-γ production (8, 36, 62). Thus, the role of T_RM_ as a source of cytokine production has important functional implications. IFN-γ is required for the protective response to chronic *T. gondii* infection, serving to activate microglia, astrocytes, and macrophages, to control parasite replication while also playing a role in T cell recruitment (5, 63–65). Overall, the CD103^+^ population has enhanced the production of critical effector cytokines and indicates that this subset of CD8^+^ T cell remains activated in the presence of ongoing inflammation unlike other subsets in the infected brain.

As expected with a T_RM_ phenotype, there is downregulation of the transcription factor *Klf2* that promotes retention within the tissue through downregulation of *S1pr1*. Our data show downregulation of the transcription factor *Klf3* in addition to *Klf2*. KLF3 has the potential to play numerous roles in a resident population. KLF3^−/−^ mice show significantly increased production of galectin-3, a mediator of diverse actions including chemotaxis and inflammation (66). Thus, the downregulation of KLF3 further supports brain CD103^+^ T cells being non-circulating or a highly pro-inflammatory population. It is clear that CD103^−^ T cells in the brain upregulate a potent array of pro- and anti-inflammatory cytokines; therefore, it would be overly simplistic to state that CD103^+^ T cells in the brain are the sole pro-inflammatory population. Nonetheless, the transcriptional data suggest that this is a primary role for this subset. An increased production of pro-inflammatory cytokines is consistent with the view that the CD103^+^ population is a T cell memory population, and increased expression of *Adam8* along with the upregulation of *Ctla4* may reflect increased TCR engagement in this subset (41, 55). Indeed, this potentially fits a model where residency is maintained by continued antigen recognition in the brain *via* dendritic cells or other APCs, and this in turn upregulates cytokine production.

An important distinction between our model and others where T_RM_ have been studied is the persistence of antigen within the brain where such antigen is continuously visible to the immune system. Previously published data using adoptive transfer of OVA-specific CD8^+^ T cells show that these cells are accumulated only in the brain when the infection was conducted with OVA-expressing parasites (36). This along with the data published here indicates that the CD103^+^ population is parasite specific. Our data are supportive of previous studies in chronic infection demonstrating exhaustion of the entire brain CD8 T cell population in the form of upregulation of several co-inhibitory receptors (*Ctla4, Pdcd1, Lag3*, and *Tigit*) relative to spleen CD103^+^ CD8 T cells (8, 36, 62). In addition, *Ctla4* is further upregulated in CD103^+^ CD8 T cells relative to other CD8 T cells in the brain, suggesting an increased engagement with antigen in this subset as alluded to previously.

The transcription factors *Hobit* and *Blimp-1* have been recently reported to be specifically required for the development of tissue-resident memory lymphocytes in the skin, gut, liver, and kidney (67, 68). Although we did not observe evidence of differential expression for these transcription factors in our data set, it is possible that the formation of tissue-resident memory T cells within the brain and other neural tissues may follow a divergent developmental pathway compared to other tissues. This could be especially true in an environment of a continuous inflammation with persistent antigen. In our data, we observe upregulation of the transcription factor *Irf4*. Expression of this gene is dependent on the strength of the initial TCR stimulus and is required for sustained proliferation and expansion of CD8 T cells in the context of influenza virus infection (69). Expression of this transcription factor by the brain CD103^+^ population could be particularly beneficial in the context of a chronic infection.

Support for the unique heterogeneity and complexity of the T cell population during chronic *T. gondii* infection is provided by the recent characterization of the T_INT_ population (9). Such cells serve as a pool from which to quickly generate effector T cells during persistent infection and serve as additional evidence that chronic infection produces unique T cell phenotypes compared to acute infections. We envision a model in which a precursor population gives rise to both T_RM_ and T_INT_ independently. Our data suggest that T_RM_ may play a significant part in the protective response to the parasite, which would be a distinct role of the T_INT_ population.

It is worthwhile to note that we have reported a substantial population of CD103^+^ CD69^-^CD8 T cells progressively accumulating in the secondary lymphoid organs that our data suggest is quiescent. Naïve CD8 T cells are capable of expressing CD103 at intermediate levels, but must downregulate CD103 upon activation (26). Although there is precedence for T_RM_ in secondary lymphoid organs during LCMV infection, such cells are uniformly CD69^+^ and do not express CD103 (21). Therefore, the accumulation of CD103^+^ CD69^−^ cells in secondary lymphoid tissue most likely represents a naïve population. Nonetheless, our data do not exclude the possibility of T_RM_ in other non-lymphoid peripheral organs. In addition to the brain, the parasite can localize to the skeletal muscle and eye during chronic phase of infection (70), and muscle-infiltrating T_REG_ populations are reported to play a critical role in tissue repair at this site (71). It is possible that T_RM_ can play a similar role during chronic infection at additional sites to the brain.

Taken together, these data provide insight into understanding the coordination of effector and memory T cell responses in the context of a chronic localized protozoan infection in the brain. We show that the expression of CD103 by brain-infiltrating CD8 T cells defines a transcriptionally distinct population during chronic *T. gondii* infection. Furthermore, we show that this population is transcriptionally consistent with tissue-resident memory T cells and has an increased capacity for pro-inflammatory cytokine production relative to other CD8 T cells in the brain. Therefore, our data indicate a need for further investigation into a unique protective role for T_RM_ during chronic infection.

## Ethics Statement

This study was conducted under Animal Use Protocol # A20140007BE with the approval of the University of California, Riverside Institutional Animal Care and Use Committee.

## Author Contributions

TL conducted all experiments. TL, SS, AR, JJ, MR, MN, IM, and EW significantly contributed to acquisition, analysis, and interpretation of data as well as revisions and final approval. TL and EW conceived and designed experiments and wrote the paper.

## Conflict of Interest Statement

The authors declare that the research was conducted in the absence of any commercial or financial relationships that could be construed as a potential conflict of interest.

## Footnotes

^1^https://github.com/tgirke/systemPipeR.

^2^http://www.bioinformatics.babraham.ac.uk/projects/fastqc/.

^3^http://www.bioinformatics.babraham.ac.uk/projects/trim_galore/.

## Supplementary Material

Figure S1 **CD103 expression kinetics and specificity of the CD8^+^ T cell subset in the peripheral secondary lymphoid organs**. **(A)** CD103^+^ CD69^+^ CD8 T cells in the spleen and lymph node. **(B)** Percentage of dextramer^+^ CD8 T cells in the spleen. Data are representative of two independent experiments with similar results.

Figure S2 **Transcriptional profile of spleen CD103^+^ population relative to spleen CD103^−^ population**. **(A)** DEG for the specified comparisons. **(B)** Venn diagram for DEG for each specified comparison. Two hundred ninety genes common to the spleen CD103^+^/spleen CD103^−^ and brain CD103^+^/spleen CD103^+^ were input into MetaCore. **(C)** Genes under categories “JAK/STAT pathway and inflammation” and “modulation of effector T cell function” were merged into one heatmap. **(D)** Heatmap of the same set of genes as in panel **(C)** for the brain CD103^+^ and spleen CD103^+^ groups. Individual replicates in heatmap were pooled from *n* = 5 mice. Values in legend are scaled values representative of reads per kilobase of transcript per million mapped reads (RPKMs). Red indicates a highly expressed gene, and blue indicates a gene with a low expression value. DEG for each comparison were determined according to the following criteria: fold change >2, FDR <5%, *p* < 0.05, and mean RPKM >1.

Figure S3 **Quantification of distance from cysts to CD8^+^ CD103^+^**. **(A)** Schematic of fields of view counted in a representative slice. **(B)** Total number of cysts versus total number of CD8^+^ CD103^+^ T cells for each field of view analyzed. **(C)** Distance to closest cyst in the field of view for each positive cell. Data are representative of two independent experiments with similar results.

Figure S4 **Genes contributing to variability in PCA plots**. **(A)** Fifty most variable genes in PC1. **(B)** Fifty most variable genes in PC2.

Figure S5 **Transcriptional profile of brain CD103^+^ CD8 T cells relative to spleen CD103^+^ CD8 T cells**. **(A)** Venn diagram illustrating unique and shared DEG for each indicated comparison. **(B)** Heatmap for a subset of the 42 DEG common to all indicated comparisons, shown in panel **(A)**. Individual replicates in heatmap were pooled from *n* = 5 mice. Values in legend are scaled values representative of reads per kilobase of transcript per million mapped reads (RPKMs). Red indicates a highly expressed gene, and blue indicates a gene with a low expression value. DEG for each comparison were determined according to the following criteria: fold change >2, FDR <5%, and mean RPKM >1.

Figure S6 **Comparison of tissue-resident memory (T_RM_) from HSV in skin to *Toxoplasma gondii***. Microarray data from the study by MacKay et al. were obtained and analyzed *via* GEO. **(A)** Venn diagram of DEG in *T. gondii* and HSV (26) for the brain T_RM_ (*n* = 3) relative to spleen T_CM_ and T_EM_ (*n* = 6). **(B)** Comparison of fold changes for the 33 DEGs common to both models. RNA-Seq DEG were determined according to the following criteria: fold change >2, FDR <5%, *p* < 0.05, and mean RPKM >1. Microarray DEG were significant if the *p* value and adjusted *p* value were less than 5%.

Figure S7 **Interaction of brain tissue-resident memory with CD11c^+^ cells in the brain**. IHC for interactions between CD103^+^ CD8 T cells and dendritic cells. **(A)** Frontal cortex, 40×. Red arrow indicates CD103^+^ CD8 T cell in contact with CD11c^+^ dendritic cell. **(B)** Zoomed image of area indicated in panel **(A)**. Representative image of *n* = 3 biological replicates. Scale bar indicates 20 µm.
