# Supplementary figures and images for: CD103+ CD8 T Cells in the Toxoplasma-Infected Brain Exhibit a Tissue-Resident Memory Transcriptional Profile
Source: Front Immunol. 2017 Mar 29;8:335. doi: 10.3389/fimmu.2017.00335 (PMC5372813; doi:10.3389/fimmu.2017.00335)

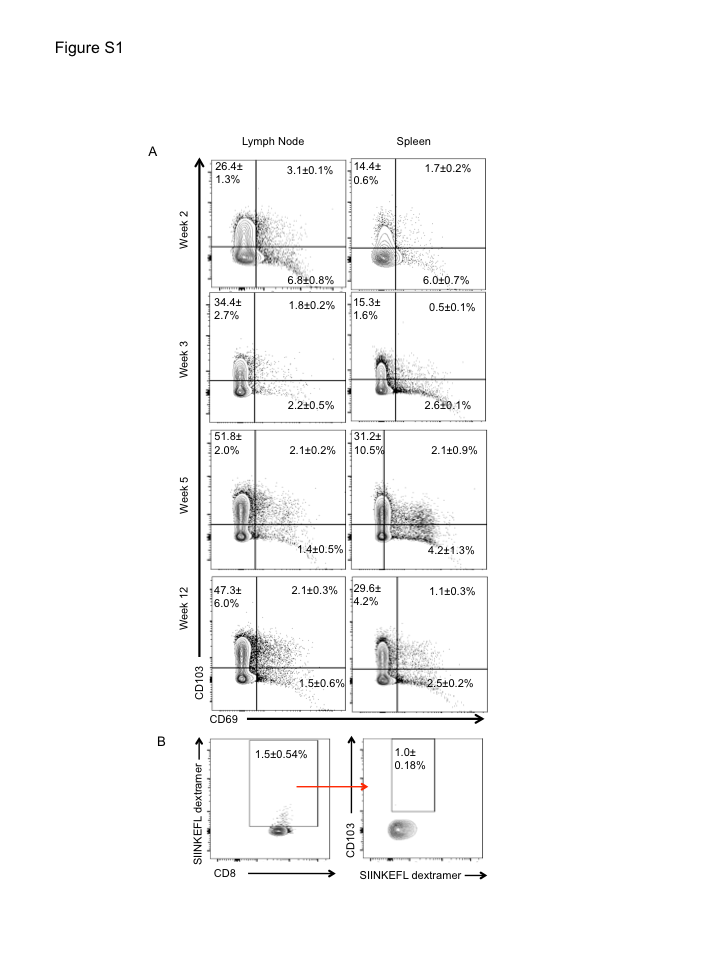

Supplement: Figure S1 — CD103 expression kinetics and specificity of the CD8+ T cell subset in the peripheral secondary lymphoid organs. (A) CD103+ CD69+ CD8 T cells in the spleen and lymph node. (B) Percentage of dextramer+ CD8 T cells in the spleen. Data are representative of two independent experiments with similar results. [file Image_1.TIFF]

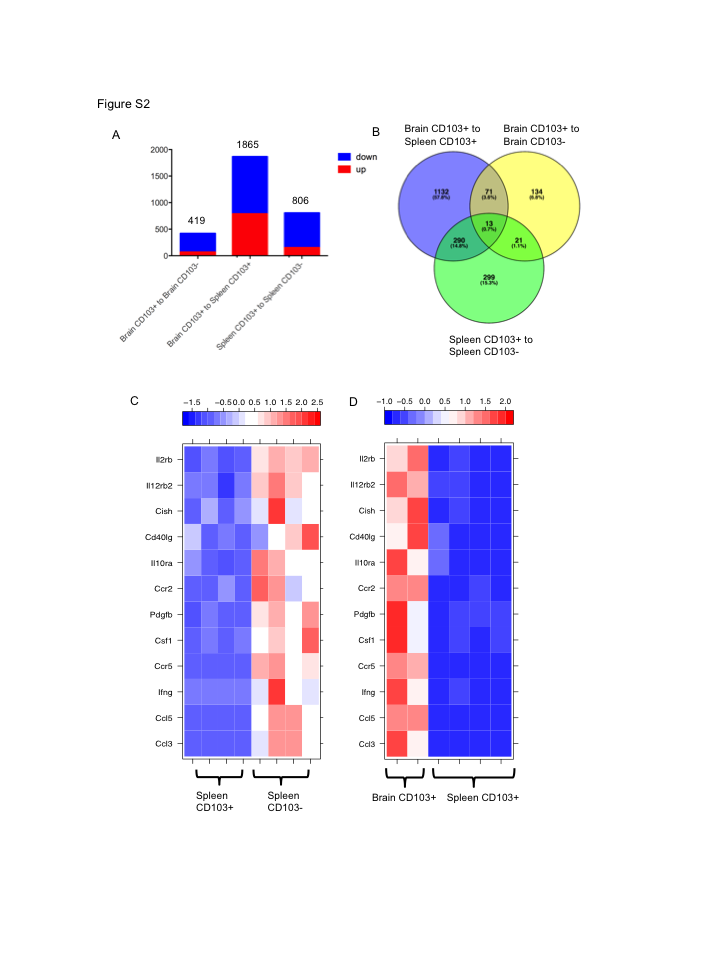

Supplement: Figure S2 — Transcriptional profile of spleen CD103+ population relative to spleen CD103− population. (A) DEG for the specified comparisons. (B) Venn diagram for DEG for each specified comparison. Two hundred ninety genes common to the spleen CD103+/spleen CD103− and brain CD103+/spleen CD103+ were input into MetaCore. (C) Genes under categories “JAK/STAT pathway and inflammation” and “modulation of effector T cell function” were merged into one heatmap. (D) Heatmap of the same set of genes as in panel (C) for the brain CD103+ and spleen CD103+ groups. Individual replicates in heatmap were pooled from n = 5 mice. Values in legend are scaled values representative of reads per kilobase of transcript per million mapped reads (RPKMs). Red indicates a highly expressed gene, and blue indicates a gene with a low expression value. DEG for each comparison were determined according to the following criteria: fold change >2, FDR <5%, p < 0.05, and mean RPKM >1. [file Image_2.TIFF]

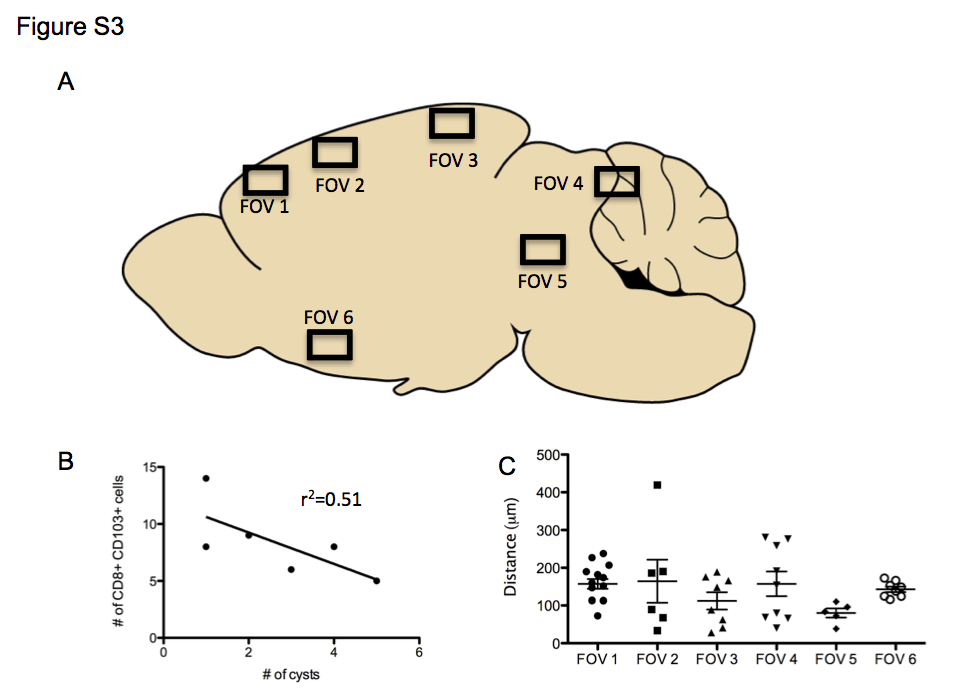

Supplement: Figure S3 — Quantification of distance from cysts to CD8+ CD103+. (A) Schematic of fields of view counted in a representative slice. (B) Total number of cysts versus total number of CD8+ CD103+ T cells for each field of view analyzed. (C) Distance to closest cyst in the field of view for each positive cell. Data are representative of two independent experiments with similar results. [file Image_3.tiff]

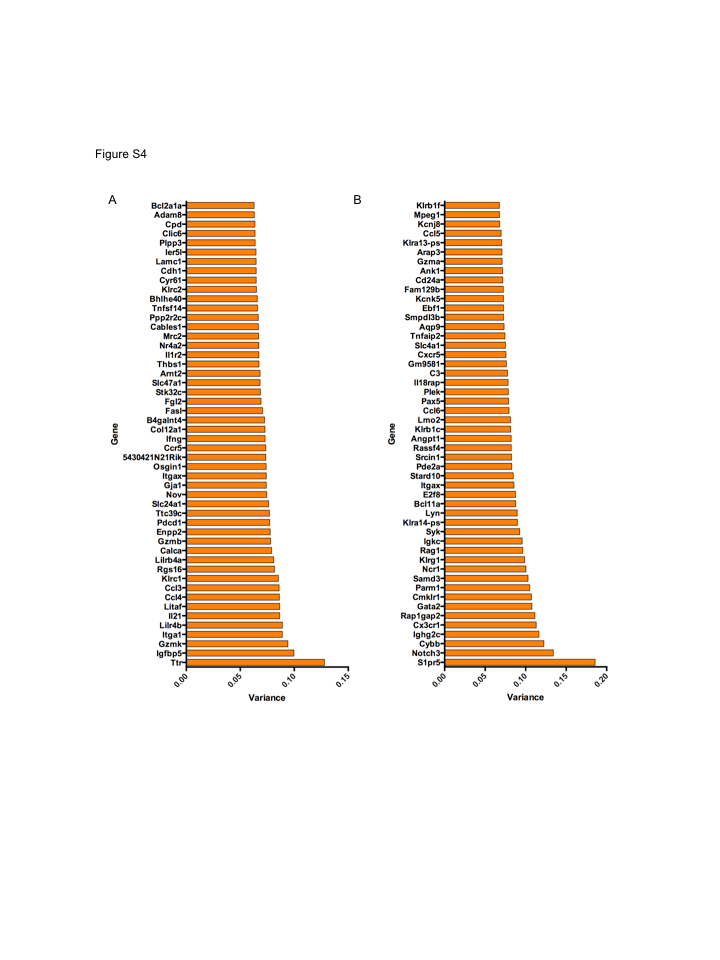

Supplement: Figure S4 — Genes contributing to variability in PCA plots. (A) Fifty most variable genes in PC1. (B) Fifty most variable genes in PC2. [file Image_4.TIFF]

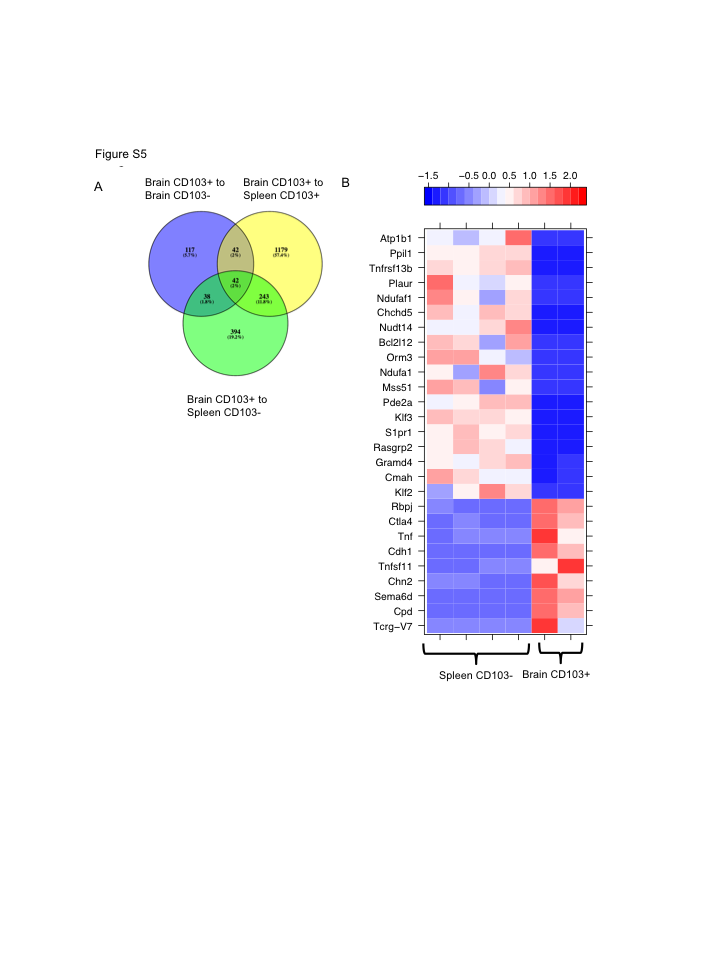

Supplement: Figure S5 — Transcriptional profile of brain CD103+ CD8 T cells relative to spleen CD103+ CD8 T cells. (A) Venn diagram illustrating unique and shared DEG for each indicated comparison. (B) Heatmap for a subset of the 42 DEG common to all indicated comparisons, shown in panel (A). Individual replicates in heatmap were pooled from n = 5 mice. Values in legend are scaled values representative of reads per kilobase of transcript per million mapped reads (RPKMs). Red indicates a highly expressed gene, and blue indicates a gene with a low expression value. DEG for each comparison were determined according to the following criteria: fold change >2, FDR <5%, and mean RPKM >1. [file Image_5.TIFF]

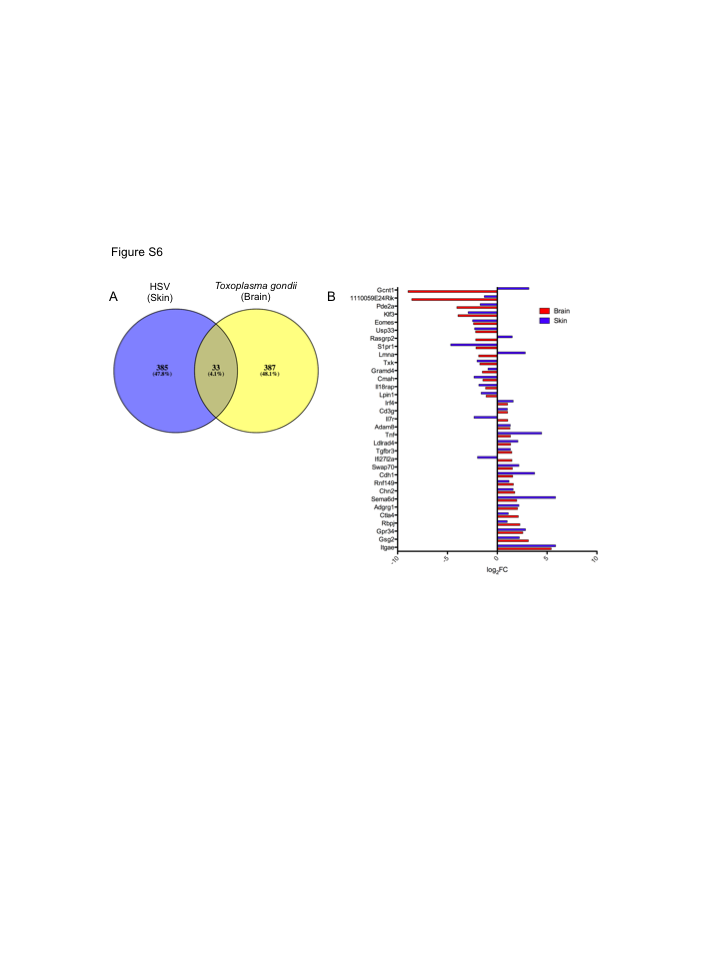

Supplement: Figure S6 — Comparison of tissue-resident memory (TRM) from HSV in skin to Toxoplasma gondii. Microarray data from the study by MacKay et al. were obtained and analyzed via GEO. (A) Venn diagram of DEG in T. gondii and HSV (26) for the brain TRM (n = 3) relative to spleen TCM and TEM (n = 6). (B) Comparison of fold changes for the 33 DEGs common to both models. RNA-Seq DEG were determined according to the following criteria: fold change >2, FDR <5%, p < 0.05, and mean RPKM >1. Microarray DEG were significant if the p value and adjusted p value were less than 5%. [file Image_6.TIFF]

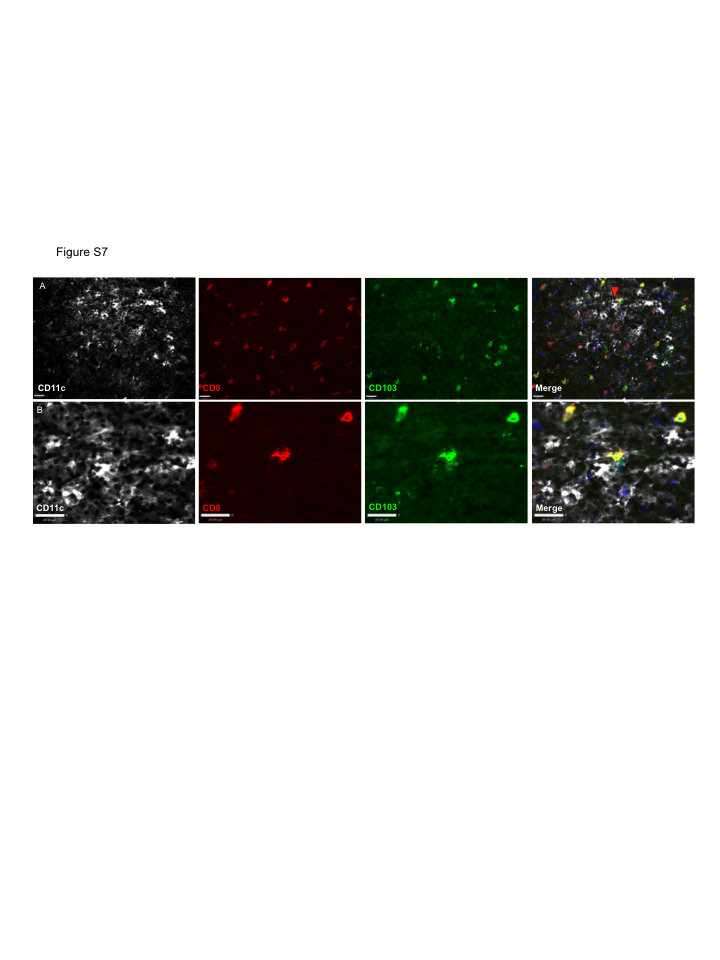

Supplement: Figure S7 — Interaction of brain tissue-resident memory with CD11c+ cells in the brain. IHC for interactions between CD103+ CD8 T cells and dendritic cells. (A) Frontal cortex, 40×. Red arrow indicates CD103+ CD8 T cell in contact with CD11c+ dendritic cell. (B) Zoomed image of area indicated in panel (A). Representative image of n = 3 biological replicates. Scale bar indicates 20 µm. [file Image_7.TIFF]
